# Use of Machine Learning for Early Detection of Maternal Cardiovascular Conditions: Retrospective Study Using Electronic Health Record Data

**DOI:** 10.2196/53091

**Published:** 2024-04-22

**Authors:** Nawar Shara, Roxanne Mirabal-Beltran, Bethany Talmadge, Noor Falah, Maryam Ahmad, Ramon Dempers, Samantha Crovatt, Steven Eisenberg, Kelley Anderson

**Affiliations:** 1 MedStar Health Research Institute Hyattesville, MD United States; 2 Georgetown-Howard Universities Center for Clinical and Translational Science Washington, DC, DC United States; 3 School of Nursing Georgetown University Washington, DC United States; 4 Invaryant Inc Roswell, GA United States

**Keywords:** machine learning, preeclampsia, cardiovascular, maternal, obstetrics, health disparities, woman, women, pregnancy, pregnant, cardiovascular, cardiovascular condition, retrospective study, electronic health record, EHR, technology, decision-making, health disparity, virtual server, thromboembolism, kidney failure, HOPE-CAT

## Abstract

**Background:**

Cardiovascular conditions (eg, cardiac and coronary conditions, hypertensive disorders of pregnancy, and cardiomyopathies) were the leading cause of maternal mortality between 2017 and 2019. The United States has the highest maternal mortality rate of any high-income nation, disproportionately impacting those who identify as non-Hispanic Black or Hispanic. Novel clinical approaches to the detection and diagnosis of cardiovascular conditions are therefore imperative. Emerging research is demonstrating that machine learning (ML) is a promising tool for detecting patients at increased risk for hypertensive disorders during pregnancy. However, additional studies are required to determine how integrating ML and big data, such as electronic health records (EHRs), can improve the identification of obstetric patients at higher risk of cardiovascular conditions.

**Objective:**

This study aimed to evaluate the capability and timing of a proprietary ML algorithm, Healthy Outcomes for all Pregnancy Experiences-Cardiovascular-Risk Assessment Technology (HOPE-CAT), to detect maternal-related cardiovascular conditions and outcomes.

**Methods:**

Retrospective data from the EHRs of a large health care system were investigated by HOPE-CAT in a virtual server environment. Deidentification of EHR data and standardization enabled HOPE-CAT to analyze data without pre-existing biases. The ML algorithm assessed risk factors selected by clinical experts in cardio-obstetrics, and the algorithm was iteratively trained using relevant literature and current standards of risk identification. After refinement of the algorithm’s learned risk factors, risk profiles were generated for every patient including a designation of standard versus high risk. The profiles were individually paired with clinical outcomes pertaining to cardiovascular pregnancy conditions and complications, wherein a delta was calculated between the date of the risk profile and the actual diagnosis or intervention in the EHR.

**Results:**

In total, 604 pregnancies resulting in birth had records or diagnoses that could be compared against the risk profile; the majority of patients identified as Black (n=482, 79.8%) and aged between 21 and 34 years (n=509, 84.4%). Preeclampsia (n=547, 90.6%) was the most common condition, followed by thromboembolism (n=16, 2.7%) and acute kidney disease or failure (n=13, 2.2%). The average delta was 56.8 (SD 69.7) days between the identification of risk factors by HOPE-CAT and the first date of diagnosis or intervention of a related condition reported in the EHR. HOPE-CAT showed the strongest performance in early risk detection of myocardial infarction at a delta of 65.7 (SD 81.4) days.

**Conclusions:**

This study provides additional evidence to support ML in obstetrical patients to enhance the early detection of cardiovascular conditions during pregnancy. ML can synthesize multiday patient presentations to enhance provider decision-making and potentially reduce maternal health disparities.

## Introduction

All other high-income nations in the world have substantially lower maternal mortality rates compared to the United States [1]. Maternal mortality rates in the United States have increased over the past 30 years [1,2], increasing approximately 85% between 2018 and 2021 [3]. There were 23.8 and 32.9 maternal deaths in the US per 100,000 live births in 2020 and 2021, respectively [4], and although the United States has one of the highest health care spending rates [5], the projected trends of maternal mortality are anticipated to continue to rise. Racial and ethnic disparities in maternal mortality rates have not only persisted, but differences in rates have widened. Non-Hispanic Black (Black) women are significantly more likely to die of pregnancy-related causes than non-Hispanic White (White) women (69.9 and 26.6 deaths per 100,000 live births, respectively) [4]. Cardiovascular conditions (eg, cardiac and coronary conditions, hypertensive disorders of pregnancy, and cardiomyopathies) were the leading cause of maternal mortality between 2017 and 2019 (27.8%) [6]. Black women have higher rates of cardiovascular morbidity and mortality than women of other races and ethnicities [7].

Programs across the United States are integrating technology to mitigate increasing maternal mortality rates, and there is a growing body of literature reporting the use of machine learning (ML) to identify patients at increased risk for hypertensive disorders in pregnancy [8,9]. Hoffman et al [8] used ML to accurately predict maternal readmission due to the complications of hypertensive disorders. ML models have also shown promising results for predicting maternal risk of hypertensive disorders of pregnancy and other cardiovascular conditions [10-12], including improved prediction accuracy when gestational age, epidemiology, hemodynamics, and biochemistry data are incorporated [9]. However, a fundamental gap remains to identify pregnant individuals at higher risk of morbidity and mortality using the power of ML and big data including electronic health records (EHRs). Therefore, this study evaluated ML technology, specifically Invaryant’s Healthy Outcomes for all Pregnancy Experiences-Cardiovascular-Risk Assessment Technology (HOPE-CAT), applied to a large health care system database, to enable early and effective risk identification of cardiovascular conditions associated with complications in pregnancy, including maternal morbidity and mortality.

## Methods

### Overview

This study was conducted using retrospective data of 32,409 obstetric patients sourced from the EHR of a large, US-based health care system with a documented birth between January 1, 2017, and December 31, 2020. This timeline was chosen to capture as many pregnancies as possible while considering factors related to when various EHR systems within MedStar Health were implemented.

### Sample

For this study, patients were selected if they met inclusion criteria: patients between the ages of 18 and 40 years at the time of the index pregnancy–related visit and who had more than 1 pregnancy-related medical encounter. To ensure the richness of the data and adequate data points to test the ability of HOPE-CAT to assess a diagnosed condition in the EHR, patients were excluded if they had limited EHR data or a single pregnancy–related medical encounter (n=11,485) and if the initial encounter within the EHR was a birth with no corresponding pregnancy data (n=14,855). This resulted in a sample of 6069 patients for analysis.

### HOPE-CAT

The ML risk assessment algorithm, HOPE-CAT, was trained via causal inference to analyze patient data and identify factors associated with the development of conditions leading to complications during pregnancy and postbirth. Clinical criteria were designated by clinical experts based on relevant literature and current standards in evaluating risk during pregnancy. Following an iterative training process, using anonymized EHRs, patient records were reviewed by HOPE-CAT on a simulated encounter-by-encounter basis. HOPE-CAT then surfaced and refined the most frequent indicators of risk within this patient population. Data analysis then validated findings against relevant data, and clinical experts then reviewed the training results and made necessary adjustments before final manual testing was conducted, and HOPE-CAT was deployed for this study. The risk factors assessed by HOPE-CAT are divided into 2 categories—static and variable (Textbox 1). Static risk factors are defined as those characteristics that do not change or change infrequently such as race, ethnicity, age, medical history, and family history. Variable risk factors are defined as characteristics that change more frequently, such as blood pressure, heart rate, and symptoms such as headache and shortness of breath.

Risk profiles were generated by patient encounters and included basic patient demographics (eg, patient ID number, age at visit, and race) and any surfaced risk factors (Textbox 1) that were noted in the patient’s record at the time of appointment. HOPE-CAT generated 2 types of risk profiles that quantify the risk factors recorded or identified in each visit: standard and high risk. High-risk, or *red flag*, profiles are defined as specific severe indicators or having 4 or more signs of risk present in a single encounter. Severe indicators that trigger a *red flag*, indicating greater risk compared to other parameters, include resting heart rate ≥120 bpm, systolic blood pressure ≥160 mm Hg, respiratory rate ≥30, oxygen saturation ≤94%, dyspnea, and orthopnea.

With this study, we primarily focused on validating the ability of the ML algorithm (HOPE-CAT) to identify pregnant individuals at high risk of cardiovascular disease within a retrospective data set. Therefore, the study design prioritized tracing outcomes for individuals flagged by the algorithm to assess its accuracy in predicting risk compared to diagnoses documented in medical records. For more extensive technical detail related to the development of HOPE-CAT, including data extraction, specifications of the computational systems, the development of the data set, and access to relevant systems, please see our previous work, Early Identification of Maternal Cardiovascular Risk Through Sourcing and Preparing Electronic Health Record Data: Machine Learning Study [10].

**Risk categories for maternal cardiovascular conditions; categories were divided into variable and static risk.**

**Symptoms (variable risk):** risk factors with no measurement scale specified were identified from the data by diagnosis codes as yes (present) or no (absent)

- Asthma, unresponsive to therapy
- Chest pain
- Dizziness or syncope
- Dyspnea (red flag risk)
- Headache, new or worsening
- Heart palpitations
- Orthopnea (red flag risk)
- Swelling of face or hands
- Tachypnea

**Physical findings (variable risk):** risk factors with no measurement scale specified were identified from the data by diagnosis codes as yes (present) or no (absent)

- Basilar crackles in lungs
- Loud heart murmur
- Oxygen saturation ≤96% (≤94% considered a red flag risk)
- Respiratory rate ≥ 24 (≥30 considered a red flag risk)
- Resting heart rate ≥ 110 beats per minute (≥ 120 beats per minute considered a red flag risk)
- Systolic blood pressure ≥ 140 mm Hg (≥160 mm Hg considered a red flag risk)

**Medical history (static risk)**

- Age (continuous in years)
- Chronic hypertension existing prior to pregnancy
- Ethnicity
- History of chemotherapy
- History of complications in labor or birth
- History of heart disease
- Prepregnancy obesity (BMI≥35)
- Pregestational diabetes
- Race
- Substance use (eg, nicotine, cocaine, alcohol, and methamphetamines)

### Ethical Considerations

Ethics approval was obtained from the Georgetown-MedStar institutional review board (STUDY00003534) and adhered to all appropriate ethical reviews and approvals, as per institutional guidelines. Institutional review board approval covered secondary analysis without additional consent. Data access, extraction, transfer, and anonymization procedures were reviewed by the institution’s data security team to ensure all necessary security requirements were implemented before the release of data. Data were deidentified before being transferred for analysis. All personal health information were removed, and patients were assigned a unique code to prevent reidentification; study identification numbers were known by a single member of the data team at the EHR institution to allow for any revalidation after original extraction. Events were also deidentified to remove any comments or free-text entry fields that could potentially be identifiable. Adherence to institutional privacy and security policies ensured patient data were protected and secured throughout the project. As this was a secondary analysis, no compensation was provided.

### Analysis

Within a virtual server environment created for this study, we systematically cleaned and standardized the EHR data (eg, organized and matched fields across tables and databases) before the deployment of an analysis by HOPE-CAT [10]. While the data set was sourced within a single health care system, standardization was necessary as data were sourced from multiple EHRs. Variables, however, were similarly defined across patients (eg, race, ethnicity, and preeclampsia). The EHR data were deidentified before analyses, enabling HOPE-CAT to truly analyze the data without consideration of race, ethnicity, or other variables that may lead to bias. Race, ethnicity, medical history, and family history were self-reported by patients and defined as recorded in the EHR.

HOPE-CAT was deployed to analyze patient data on an encounter-by-encounter basis, as in each visit and data collection on record were analyzed in the order of entry. Data from each encounter included patient demographics, physical findings, symptoms, and medical history and were analyzed by HOPE-CAT to identify changes and trends. In instances where HOPE-CAT detected any sign of risk, based on the training criteria, a risk profile was generated for the given patient and encounter [10]. Due to the nature of the available data, it was not possible to determine whether a patient’s pregnancy was their first. Each pregnancy was analyzed in isolation, and risk profiles were only compared to outcomes within that individual pregnancy.

Following full analysis by HOPE-CAT, patient risk profiles were linked to any outcomes of cardiovascular pregnancy conditions and complications, specifically preeclampsia and eclampsia, cardiomyopathy, myocardial infarction (MI), heart failure, acute kidney disease and failure, cerebral infarction, pulmonary embolism, venous thromboembolism, and HELLP (hemolysis, elevated liver enzymes, and low platelets) syndrome. Once a risk profile was identified and linked with a patient’s outcomes as stated in the EHR, a difference was calculated based on the date of the risk profile compared to the date of diagnosis or intervention recorded in the patient’s records. For example, if HOPE-CAT detected a risk that indicated a patient may be experiencing symptoms of preeclampsia on day 114 and the patient’s EHR data showed a diagnosis of preeclampsia on day 142, the difference would be 28 days. In-depth manual reviews were conducted by retrieving the relevant data against the results of HOPE-CAT and then cross-checking and tabulating each result. The tabulated information was further cross-checked by the independent quality team.

## Results

**Figure 1:**
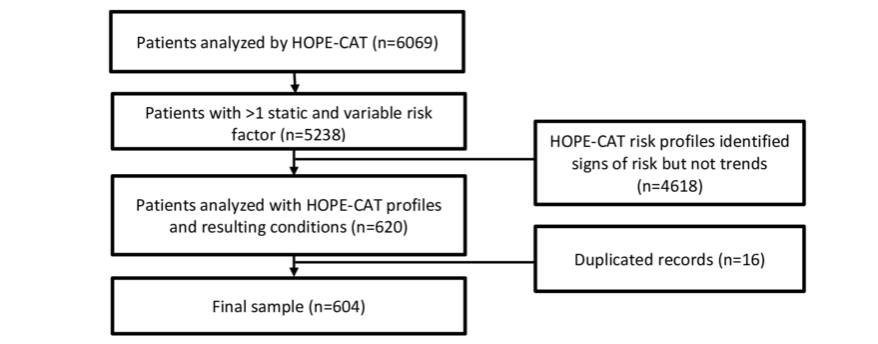
Flow diagram showing sample selection process for HOPE-CAT (Healthy Outcomes for all Pregnancy Experiences-Cardiovascular-Risk Assessment Technology) analysis.

Of the 6069 patients analyzed by HOPE-CAT, 5238 patients had 1 or more risk factors (Figure 1). A total of 1716 (32.8%) *red flag* risk profiles were identified among patients with 1 or more risk factors. Of the 1716 *red flag* risk profiles developed, HOPE-CAT identified risk profiles for 620 patients who could be matched with outcomes of interest in the patient’s diagnosis and intervention (medication) data. There were patients in the final subset for which HOPE-CAT identified risk of cardiovascular conditions after a diagnosis was made (ie, the delta was a negative number). These patients were included for accurate representation.

Following the identification of all resulting risk profiles, 16 patients who were identified by HOPE-CAT in duplicate (eg, risks that tracked to 2 different outcomes) were combined. This resulted in a final sample of 604 patients for whom available records of diagnoses or interventions could be compared against and linked with the identified risk profiles.

The majority of the final sample self-identified as Black (n=482, 79.8%) and between the ages of 20 and 34 years (n=509, 84.4%; Table 1). Twenty-one patients had 2 pregnancies recorded during the study period.

Preeclampsia was the most common condition diagnosed in our sample (n=547, 90.6%), followed by thromboembolism (n=16, 2.7%) and acute kidney disease or failure (n=13, 2.2%; Table 2).

The average delta for the final subset of 604 patients was 56.8 (SD 69.7) days between when HOPE-CAT pinpointed risk factors during a patient’s visit and the first date of diagnosis or intervention of a related condition in the patient’s record. For patients who were diagnosed with preeclampsia, HOPE-CAT identified risk factors an average of 60.2 (SD 90.9) days earlier than the time point indicated in the records. Patients with a diagnosis of MI were identified with risk factors for MI on average 65.7 (SD 81.4) days earlier than the first reported date of diagnosis. For patients whose pregnancy experience resulted in cerebral infarction (n=4, 0.7%), the delta was 42.3 days. Of these 604 patients, 19 (3.1%) experienced 2 or more tracked conditions (eg, preeclampsia with acute kidney failure and peripartum cardiomyopathy).

**Table 1:** Participant demographics of race, ethnicity, and age in years (N=604).

Characteristic			Values, n (%)
**Race^a^**			
	American Indian or Alaska Native	1 (0.2)	
	Asian	1 (0.2)	
	Black	482 (79.8)	
	White	86 (14.2)	
	None of the above or unknown race	34 (5.6)	
**Ethnicity**			
	Hispanic	7 (1.2)	
	Non-Hispanic	597 (98.8)	
**Age (years)**			
	18-19	1 (0.17)	
	20-34	509 (84.4)	
	35-40	94 (15.6)	

^a^Self-identification as reported in the electronic health record.

**Table 2:** Patient subset by condition identified in the electronic health record, including delta (N=604).

Conditions	Patients, n (%^a^)	Delta mean (SD) (days)
Preeclampsia	547 (90.6)	60.2 (90.9)
Thromboembolism	16 (2.7)	15.3 (37.7)
Acute kidney disease and failure	13 (2.2)	25.1 (43.2)
Cardiomyopathy	10 (1.7)	13.9 (43.9)
Eclampsia	9 (1.3)	46.2 (58.3)
HELLP^b^ syndrome	7 (1.2)	34.0 (34.8)
Heart failure	5 (0.8)	13.6 (13.2)
Cerebral infarction	4 (0.7)	42.3 (36.8)
Myocardial infarction	3 (0.5)	65.7 (81.4)

^a^Percentage of patients does not add to 100 as some patients had multiple conditions.

^b^HELLP: hemolysis, elevated liver enzymes, and low platelets.

## Discussion

### Principal Findings

In this study, high-risk cardiovascular conditions in pregnancy were identified earlier than determined by a health care provider by leveraging Invaryant’s HOPE-CAT ML technology to surface signals and trends in patients’ medical records. HOPE-CAT enabled early and effective screening of potential risks from cardiovascular disease an average of 56.8 (SD 69.7) days earlier than the first date of diagnosis or intervention of a related condition as documented in the EHR.

Cardiovascular disease is the leading cause of death for women in the United States, and overall, US women experience challenges in cardiovascular care, with notable underdiagnosis and late presentations, making it less likely for them to receive appropriate treatments [13]. Black women, specifically, experience cardiovascular disease at a higher rate than White women [14,15] and are also significantly more likely to die of pregnancy-related causes [4]. Pregnancy-related disorders are not only associated with complications during pregnancy, but they also portend future cardiometabolic and long-term cardiovascular-related morbidity [13]. Implicit racial biases contribute to these health inequities, resulting in increased maternal morbidity and mortality when Black women with valid and important health concerns are dismissed [16]. Enabling early and effective screening for pre-existing comorbidities and early identification of risk with enhanced technological applications, like HOPE-CAT, independent of patient characteristics and descriptors or provider bias [17], has the potential to mitigate factors leading to racial biases [18]. Screening is imperative to promote optimal cardiovascular health to generate appropriate referrals and collaborations among health care specialties [19]. Cardio-obstetrics teams have demonstrated some promising outcomes in women with known cardiovascular disease, and early identification may facilitate the inclusion of patients who are pregnant into these multidisciplinary care teams in a timely manner [20].

HOPE-CAT also offers an important clinical tool for providers to enhance the early detection and intervention of cardiovascular disease in pregnancy by facilitating the delivery of care based on trends and synthesis of data over time rather than only the current presentation of the patient at a single visit. The ability to create risk profiles dependent on patient presentation and independent of provider recall may improve the accuracy of disease identification and promote changes in provider recommendations, monitoring, treatment, referrals, and even patients’ self-monitoring and awareness of risk. Importantly, HOPE-CAT generates a risk profile based on multiple factors that are routinely collected in prenatal visits. This approach has been argued to be superior to prediction models that require information not routinely collected [21] or to “static and single-class conventional prediction methods” in the detection of hypertensive disorders in pregnancy and post partum [9]. A real benefit of HOPE-CAT lies in its deployment in areas with limited resources and providers without obstetrical specializations. Early identification of pregnant individuals at high risk for cardiovascular disease would allow more prompt referral to high-risk clinics and specialist care.

Finally, identifying and treating cardiovascular diseases early in a pregnancy can alleviate stress on health care systems including decreasing costs of hospitalization and urgent care, and more importantly, decreasing the risk of morbidity and mortality among expecting mothers. Maternal mortality affects a country’s economic well-being. The total cost of US maternal mortality in 2019 was estimated to be US $32.3 billion from birth to the child reaching their fifth birthday [22]. In the United States, maternal mortality has been rising since 2000, even with respect to gross domestic product and health expenditure per capita [23]. It should be noted that while ML technology is a valuable tool for providers in the care of birthing persons resulting in decreased costs in the long term, the possibility exists that there could be additional short-term costs related to testing and interventions [8].

### Comparison With Prior Work

Our retrospective study adds to the body of research exploring the use of ML in clinical practice by leveraging the power of EHR data to evaluate HOPE-CAT’s early identification of cardiovascular risk. This study also adds to the limited ML research in the field of obstetrics. A review conducted between 2000 and 2018 including 386 studies reporting on the use of ML in clinical practice found only 10 studies focused specifically on the field of obstetrics and gynecology [24]. Another systematic review analyzed publications on the use of ML application in obstetrics and gynecology core discipline journals and found only 19 publications between 2000 and 2020 [25].

In obstetrics, ML has successfully been used to determine the clinical parameters most useful for predicting preeclampsia and hypertensive disorders of pregnancy [12,26,27]. Our study provides additional evidence to support the use of ML in the field of obstetrics. Unlike prior work, our purpose was not to determine which parameters were most predictive but rather how soon HOPE-CAT could determine risk, based on an iterative, encounter-by-encounter basis [28]. Specifically, this study offers a novel tool for monitoring cardiovascular risk during pregnancy using real-time trends, thereby assisting health care professionals in the provision of perinatal care for high-risk patients. Consistent with earlier inquiries on the deployment of ML within obstetrics, our ML model has undergone technical validation [10]. Nonetheless, the imperative remains for further research to establish the clinical validity of the model.

### Limitations

This study had several limitations. Complete EHRs were not always available for proper analysis and we did not have complete access to patients’ full health history [29]. Compared other studies applying ML to big data [27], we relied on EHRs and did not include other types of unstructured data (eg, clinical notes) in this analysis. This may have created limitations when interpreting results. In order to optimize the performance of HOPE-CAT, several steps were required to clean and standardize the data set due to sourcing from multiple EHRs. This does not negate the validity or capability of the ML technology but does describe the nonstandardized, noninteroperable nature of EHRs data in the present day. The retrospective nature of our study was limited to data available within the health system’s EHRs and has been identified as a limitation in many prior studies using ML [24]. However, the advantage of retrospective data is that it allows for in-depth manual reviews. The data for this study were collected between January 1, 2017, and December 31, 2020. An overlap with the COVID-19 pandemic may have resulted in skewed data; pregnant individuals may have been reluctant to seek care or faced additional barriers to accessing care in 2020, which may have resulted in delayed diagnosis of complications. Additionally, analysis by race, ethnicity, or age was not possible with our sample, and thus we may have failed to capture important differences, such as whether the average delta may have been different by ethnicity, race, or age. Finally, this study was conducted within 1 health care system, and the results may not be generalizable to a different demographic or setting.

### Conclusions

The findings from this study provide the foundation for future work to evaluate ML prospectively, in vivo, in the real-world setting, and longitudinally during current pregnancies and to inform future pregnancies, postpartum events, and overall cardiovascular health. Future ML may integrate multiple data sources including unstructured data using natural language processing, wearables, remote patient monitoring devices (eg, blood pressure), and symptom surveys. Additionally, the integration of factors related to social determinants of health may inform solutions to advance health equity and address the increasing US maternal mortality rates that show widening disparities associated with race and ethnicity. To facilitate the reversal of this trend, it is imperative that risk identification occurs earlier in the pregnancy trajectory to allow for increased monitoring and referral to more specialized care [30] and that ML technology is leveraged to support maternal health screening in routine appointments. The results from this study of ML through HOPE-CAT provide foundational evidence to develop solutions to mitigate the harmful impacts of pregnancy and improve maternal health for all.

## Abbreviations

bpm: beats per minute
EHR: electronic health record
HELLP: hemolysis, elevated liver enzymes, and low platelet
HOPE-CAT: Healthy Outcomes for all Pregnancy Experiences-Cardiovascular-Risk Assessment Technology
MI: myocardial infarction
ML: machine learning

## Data Availability

The data sets generated and analyzed during this study are available from the corresponding author upon reasonable request.
